# Degenerative Cervical Myelopathy: A Brief Review of Past Perspectives, Present Developments, and Future Directions

**DOI:** 10.3390/jcm9020535

**Published:** 2020-02-16

**Authors:** Aria Nouri, Joseph S. Cheng, Benjamin Davies, Mark Kotter, Karl Schaller, Enrico Tessitore

**Affiliations:** 1 Department of Neurosurgery, University of Geneva, 1205 Geneva, Switzerland; Karl.Schaller@hcuge.ch (K.S.); enrico.tessitore@hcuge.ch (E.T.); 2 Department of Neurosurgery, University of Cincinnati College of Medicine, Cincinnati, OH 45267-0515, USA; chengj6@ucmail.uc.edu; 3 Department of Clinical Neurosciences, University of Cambridge, Cambridge CB2 0QQ, UK; bd375@cam.ac.uk (B.D.); mrk25@cam.ac.uk (M.K.)

**Keywords:** focus issue, update, cervical spondylotic myelopathy, compressive myelopathy

## Abstract

Degenerative cervical myelopathy (DCM) is the most common cause of spinal cord injury in developed countries; its prevalence is increasing due to the ageing of the population. DCM causes neurological dysfunction and is a significant cause of disability in the elderly. It has important negative impacts on the quality of life of those affected, as well as on their caregivers. DCM is triggered by a variety of degenerative changes in the neck, which affect one or more anatomical structures, including intervertebral discs, vertebrae, and spinal canal ligaments. These changes can also lead to structural abnormalities, leading to alterations in alignment, mobility, and stability. The principle unifying problem in this disease, regardless of the types of changes present, is injury to the spinal cord due to compression by static and/or dynamic forces. This review is partitioned into three segments that focus on key elements of the past, the present, and the future in the field, which serve to introduce the focus issue on “Degenerative Cervical Myelopathy and the Aging Spine”. Emerging from this review is that tremendous progress has been made in the field, particularly in recent years, and that there are exciting possibilities for further advancements of patient care.

## 1. Introduction

Degenerative cervical myelopathy (DCM) is a broad term, representing the various age-related degenerative conditions of the cervical spine that result in neurological injury to the spinal cord through static and dynamic injury mechanisms. The term DCM was introduced in 2015 in an effort to standardize the terminology [1], provide a clear definition, and provide an outline of conditions that fall under this term.

**Figure 1:**
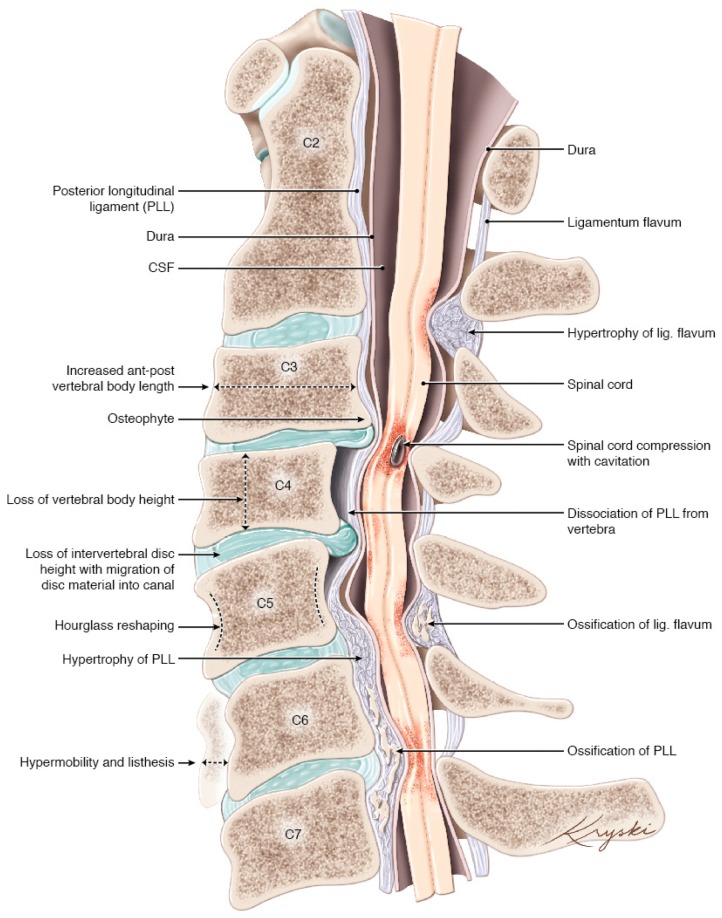
Artistic depiction of the various degenerative changes that can be seen in patients with DCM (Concept Aria Nouri, edits Michael G. Fehlings, artwork design Diana Kryski). CSF = Cerebrospinal Fluid, PLL = Posterior Longitudinal Ligament. Originally published in Nouri et al. *Degenerative Cervical Myelopathy: Epidemiology, Genetics and Pathogenesis. Spine (Phila Pa 1976). 2015;40(12): E675-93*.

The pathogenesis of cervical spine degeneration often progresses in the following manner: general degenerative changes of the spine begin at the disc. With age, the disc becomes less compliant, principally due to a reduction in water content and fibrosis of the nucleus pulposus. This process results in the loss of the ability of the discs to distribute pressure forces equally onto the vertebral endplates. Bone remodeling at the endplates creates osteophytes and changes in the structure of vertebrae. As this process is likely triggered by pressure forces over time, the degenerative evolution may reflect a function of age and use intensity. Other alterations that occur during this process include a loss of disc and vertebral height, resulting in the in-folding of the ligamentum flavum, which may also hypertrophy in response. As a consequence of these anatomical changes, cervical alignment changes, spondylolisthesis, and hypermobility may develop. This can occur at single or multiple levels. These changes may also potentially stimulate ossification of the spinal ligaments; however, the occurrence and propensity of ossification are probably influenced by genetic factors [1,2,3] (Figure 1). These changes are most commonly incidental, and do not manifest symptoms, however, in some, they may become sufficient to cause spinal cord injury through static compression of the cord, dynamic injury through instability, cord stretching due to tethering, or a combination of these factors [1].

This initiates a cascade of secondary injury events within the spinal cord, including ischemia, inflammation, and apoptosis, that results in cervical myelopathy [4]. Like the degenerative pathology that causes it, the symptoms are also variable. Commonly, they can include loss of digital dexterity, weakness, imbalance and frequent falls, sensory loss, pain, and/or bladder or bowel dysfunction, in most severe cases. Together, this syndrome comprises the most common cause of spinal cord injury in the developed world.

This review will highlight some important elements of the history of DCM, classically called cervical spondylotic myelopathy (CSM), the present status, and interesting future directions. Together, this discussion serves as an introduction to the focus issue “Degenerative Cervical Myelopathy and the Aging Spine”.

## 2. The Past

### 2.1. Transition from CSM to DCM

The term CSM has been used to describe vertebral degenerative disease that cause myelopathy; however, it lacked a formal and unifying definition, which has created a number of challenges. The term “spondylosis” probably came from spondylosis deformans, which itself derived from spondylitis deformans likely coined by Rokitansky in 1844 [5], mentioned by Beneke in 1897 [6], and popularized by Schmorl in 1931 [7]. The change from spondylitis to spondylosis likely arose from the distinction that spondylitis represents an infectious process, whereas spondylosis represents a degenerative process. Francois et al. (1995) discussed this history in a paper that brought together a study group from the Committee of Pathology of the European League against Rheumatism [5]. Therein, the group stated that there is no agreement on the term “spondylosis”, that the group recommended avoidance of the term, and that it should be defined whenever used.

Despite this recommendation, CSM has continued to be used but variably defined. For example, ossification of the posterior longitudinal ligament (OPLL) is considered by some a subtype of CSM, and others a distinct pathology [8]. This has proved a challenge for literature synthesis [9] and has consequently hindered many important lines of investigation, including the evaluation of prevalence rates for specific phenotypes, risk factors for disease development, the natural history, and surgical decision-making. It is also possible that the inconsistent and complex terminology has contributed to a lack of disease awareness [10], which has been considered to be a contributing factor to diagnostic delay and disability [11]. 

Recognizing these issues, a new term, “Degenerative Cervical Myelopathy”, was proposed and defined in a paper in 2015 [1]. The term encompasses both CSM and OPLL and more clearly recognizes the degenerative nature of the disease and its association with advanced age. 

The transition from CSM to DCM is ongoing. However, its increasingly widespread adoption, including in the treatment guidelines by AO Spine and an ongoing research efficiency initiative [12], is indicative of its requirement and acceptance [13]. 

### 2.2. Prevention of Neurological Decline to Recovery of Neurological Function

In the past, surgical treatment was recommended to arrest further neurological decline. Historically, many even considered it a last resort. However, it has become apparent, mostly in the last decade, that many patients with DCM not only stop declining in neurological function but also may improve: in two of the largest prospective observational studies, the average improvement of neurological function based on the mJOA (modified Japanese Orthopedic Association) scale was between 2–3 [14,15]. This clarification has fundamentally altered practice, in that patients with mild myelopathy or with a stable condition may now be offered surgery, whereas before this may not have been the case. Likewise, this has also changed the counseling of patients with regards to the benefits and risks of surgery intervention.

Furthermore, there is growing evidence in the literature concerning the need for identifying patients who may benefit more from surgery [16,17]. Indeed, appropriate patient selection may lead to better surgical results, and, as a consequence, to a better appreciation of surgery among DCM patients.

Recent research, including that featured in the present special issue, indicates that improvements can occur irrespective of age. While some data indicate that old age is a predictor of a less beneficial outcome, it has consistently been shown that the elderly population is also capable of neurological recovery [18,19,20].

A remaining knowledge gap surrounds mild, stable myelopathy or spinal cord compression without clear or typical signs of myelopathy. Recent guidelines on this subject have indicated that patients with asymptomatic spinal cord compression should not be recommended surgery but should undergo a close clinical follow-up, and that patients with mild myelopathy should be offered surgical intervention or supervised structured rehabilitation [13]. These consensus statements are largely based on expert opinion, with limited empirical data to draw upon, owing to current practice conventions. However, the significant impact of surgery identified in these recent studies, aligned with the recognition that timely treatment is critical to recovery, may in the future see further paradigm changes.

### 2.3. Cervical Canal Stenosis to Cervical Cord-Canal Mismatch

The presence of a narrow canal has been widely considered to be a risk factor for the development of DCM [21]. However, the evidence is limited and largely based on data from acute traumatic spinal cord injury. This predisposition has been called congenital cervical stenosis, or developmental stenosis, or developmentally narrow canal, and measurements for this was classically done using the Torg–Pavlov ratio (TPR) or an absolute canal diameter (sometimes also called “space available for the cord” [22]) [23,24]. However, the terms used are not entirely accurate, whilst the TPR has been outdated. There may be some conditions that create a congenital stenosis, such as in achondroplasia [25], but it is unclear whether canal parameters remain the same throughout development into adulthood. Furthermore, recent studies demonstrating the measurements of normal canal and cord parameters have indicated that both the size of the canal (small canal) and the cord (large spinal cord) can predispose to the development of DCM [26]. As a consequence, spinal cord occupancy ratio (SCOR) was developed to account for both factors accurately and to enable risk determination for DCM development [27]. It has also been proposed that perhaps a cord-canal mismatch may be a better term, and that a SCOR defined as ≥70% on midsagittal imaging or ≥80% on axial imaging appears to be an effective method of identifying cord-canal mismatch [21,28,29].

Classical diagnostic criteria for diagnosing “congenital stenosis” include TPR of 0.80–0.82 [23,24] and an absolute canal diameter of 12–13 mm [30,31].

## 3. The Present

### 3.1. Current Population Trends and Epidemiology

Whilst it is well accepted that DCM is the most common cause of spinal cord injury in the developed world, the epidemiology of DCM remains poorly characterized. In North America, the incidence and prevalence was estimated at a minimum of 4.1 and 60.5 per 100,000, respectively [1]. In Taiwan, a population-based study reported a hospitalization of 4.04/100,000 person-years [32], and in the Netherlands, an incidence based on a fixed referral system of 1.6/100,000 inhabitants was reported [33]. Published studies also report that males are more commonly affected than females.

However, it is anticipated that these are significant underestimates, as they rely on operative incidence (which fails to account for the likely larger non-operative cohort) and will not account for widespread underdiagnosis. Anecdotal insights are provided by MRI series of ‘healthy volunteers’, which have identified an age-associated prevalence of spinal cord compression; for example, in one series of randomly selected volunteers aged 40–80, incidental cervical cord compression was detected on MRI in 59% of individuals (108/183, ranging from 31.6% in the fifth decade to 66.8% in the eighth decade) [34]. Two (1%) were found to have incidental DCM. Additionally, patients with canal stenosis or non-myelopathic compression of the spinal cord are at risk of DCM development, with those developing DCM estimated to be approximately 8% at 1-year follow-up and 23% at a median of 44-months follow-up [35]. Taken together, this data points to a significant and hidden disease burden.

The association with age aligns with emerging evidence that surgical rates for DCM are rising, particularly cervical spine fusion procedure [36,37,38]. It would seem that with an aging population and increased recognition of the prevalence of DCM that this trend will continue to rise. Along with this, a rise in cost and global burden should be anticipated [39].

Based on a global cohort of patients derived from the multicenter AO Spine studies on DCM, most patients present with multi-level degeneration (spondylosis) and more than 50% have accompanying ligamentum hypertrophy or in-folding that is contributing to this compression. OPLL was shown to be present in about 10% of patients, with a significantly higher prevalence in Asia [40].

### 3.2. Debate: Anterior vs. Posterior Surgical Approach

Decompression of the spinal cord and stabilization of the spine can be achieved safely from both anterior and posterior approaches. Furthermore, multiple options exist for either of these paths. Of course, a combined approach is also an option, but is usually reserved for the most severe cases. The relative merits of these approaches have been, and continue to be, a hot topic for debate amongst surgeons, as each approach carries differing risk profiles and healthcare costs. A recent propensity-score matched study on inpatient complications on 13,884 (*n* = 6942 ACDF [Anterior Cervical Decompression and Fusion]; *n* = 6942 PCDF [Posterior Cervical Decompression and Fusion]) patients demonstrated PCDF to be associated with greater length of stay, in-hospital costs, and general medical and surgical complications, while ACDF carried higher risk of postoperative hematoma, hoarseness, and dysphagia [41]. However, an MRI-based propensity-score-matched analysis comparing anterior vs. posterior surgery showed no significant differences in neurological outcome [42]. It is anticipated that a randomized multicenter prospective study (CSM-S trial, identifier: NCT02076113) will provide some final clarity on a subject matter that has predominated the DCM literature over the last decade [43].

Equivalence of outcome in patient populations does not mean that this also holds true at the individual level. The authors advocate a patient-tailored approach that considers individual nuances of the preexisting pathology for surgical planning; not all patients are amenable to both approaches, and management of deformity and surgical preference, as well as expertise, are important factors to consider. Indeed, this has been echoed in a global survey of spine surgeons [44], which indicated that a significant anterior cord compression and cervical kyphosis strongly influenced surgeons toward an anterior approach, whereas a high degree of posterior cord compression, congenital stenosis, and multilevel compression or OPLL were the strongest factors influencing surgeons toward a posterior surgery. 

Indeed, cervical alignment has become a focus of surgical research in recent years. Baseline radiological measures including C2-7 angle, C2-7 sagittal vertical axis, T1 slope, and modified K line have been proposed to support anterior vs. posterior decision-making. For example, kyphosis exceeding 13°, based on a C2–C7 angle, has been proposed to require anterior decompression or correction of kyphosis in addition to posterior decompression [45]. Others have suggested that a high T1 slope is a risk for kyphosis development in patients with laminoplasty [46]. A more recently described measurement, the modified K-line, represents a line connecting the midpoints of the spinal cord between C2 and C7 on midsagittal MRI, which is then used to measure the minimum interval distance between the line and anterior compressive factors. Using this measurement, the authors suggest that a minimum of 4.4 mm of space between the K-line and the anterior compressive border is related with an optimal neurological recovery in non-lordotic patients after laminoplasty [47]. Taken collectively, these studies indicate alignment should be an important component of surgical decision making. However, these studies demonstrate clinical outliers, indicating that these radiological algorithms do not fit all cases and that such measurements should represent only part of the decision-making process.

### 3.3. Engaging Patients through Advocacy and Knowledge Dissemination

Despite being common, there is lack of recognition with regards to DCM. The reasons for this may be multifactorial; likely contributing factors include the mentioned lack of a clear definition of CSM, the lack of advocacy or patient support, and a lack of recognition among the general medical community and beyond. However, efforts are underway to tackle this. As mentioned earlier, a new terminology has been introduced to address the lack of definition and unify the field. Furthermore, RECODE-DCM (REsearch objectives and COmmon Data Elements for DCM, https://www.recode-dcm.com) an international consensus process involving all important stakeholders (people with myelopathy, carers, surgeons, and other healthcare professionals) to (1) identify key knowledge gaps in the field, (2) define a core outcome set and data elements for DCM, and (3) reach agreement on a unifying term [12]. Moreover, we have established Myelopathy.org, the first charity for promoting DCM (Cambridge, UK). It hosts an international peer-to-peer support community and aims to improve awareness and outcomes for patients by bringing together DCM stakeholders internationally (https://myelopathy.org/).

## 4. The Future

### 4.1. Multi-Dimensional Approach to Surgical Outcome Prediction

Over the last few years, there has been a growing recognition that certain factors are able to predict surgical outcomes in patients with DCM. Prediction models using both MRI and clinical data have been developed using the global multicenter data from the AO Spine prospective studies on the effectiveness of surgery for DCM (for example, see [16,48]). However, these studies have shown that there are limits in the predictive capacity of the models. Elements that may be able to improve the current prediction models but have not yet been tested include advanced MRI and electrophysiology. Both advanced MRI measures, such as fractional anisotropy (DTI) [49], grey matter to white matter ratio (T2 *) [50], and electrophysiology [51], are, to an extent, capable of predicting surgical outcome. A novel generation of models that include such measures may improve the ability to predict surgical outcome [17]. Furthermore, machine learning techniques may also facilitate improved predictive capacities [52]. Specifically, they may provide another dimension of data analysis. However, further research is required to better understand how to utilize such techniques.

### 4.2. Transcranial Magnetic Stimulation (TMS) and DCM: Assessing Cortical Volume and Function

Little is known with regards to cerebral changes that occur in patients with DCM. Given that DCM patients lose physical functions, a few studies have now started to consider cortical correlates. One such recent study using TMS, a method by which a magnetic field is used to noninvasively stimulate a region of the brain, has proposed the concept of a “corticospinal reserve capacity”, having identified a decrease in the cortical motor area activity and a compensatory increase in supplementary motor area activity in patients with DCM [53]. The potential implications of this concept may be significant. Does it help to explain the frequently observed discordance between clinical findings and those seen on MRI? MRI findings correlate weakly with baseline severity and outcome, and in fact patients with little compression can be disproportionately affected with regards to symptoms, whereas other patients with severe compression on imaging are relatively mildly affected with regards to their symptoms [54,55]. Indeed, differences in corticospinal reserve capacity may explain this phenomenon. Moreover, the finding, and the manner of its identification, hold therapeutic implications—is it possible, for example, to stimulate the deficient motor areas via TMS to recuperate motor function? Time will have to address this, as this area remains currently largely unexplored.

### 4.3. A Wave of New Biomarkers

At the present time, DCM remains a clinical diagnosis, which is confirmed via imaging, typically MRI, and sometimes with the aid of electrophysiology. Having additional biomarkers would benefit the diagnosis of DCM, and as such, this is the subject of ongoing work including advanced MRI and machine learning techniques, as outlined above. For example, quantitative MRI techniques such as grey to white matter ratio and fractional anisotropy may be able to detect progression of myelopathy severity more sensitively than classical measures such as the mJOA [56]. On the other hand, electrophysiology has been used for some time in DCM to aid diagnosis and for intraoperative monitoring purposes, but there remains significant room for improvement in this area. Some research has shown both the diagnostic and prognostic value of electrophysiology [51], however, electrophysiology has not been fully integrated into clinical practice, with the exception of its use for neuromonitoring.

Outside of imaging and electrophysiology, there are no CSF or blood biomarkers available to aid with diagnosis. However, work in this area is ongoing [57], and it is conceivable that biomarkers will be available in the future to aid in the diagnostic process. Some of this research has been done in the realm of acute spinal cord injury, which may be translatable to DCM [58], and some preliminary work has been done in DCM, such as with miR-RNA-21, miR-34a for, and miR-10a, which have been linked to neuroinflammation, neuronal apoptosis, and OPLL, respectively [59,60,61].

## 5. Conclusions

The last decade has seen significant scientific advances in DCM, driven by high quality studies in surgical management and supported by the attempt to standardize the nomenclature, including an index term. However, these studies have also demonstrated the extensive residual disability of patients after surgery and the requirement for further progress.

It is clear that an emerging goal of future research is to inform timely treatment on an individual basis, not only to arrest clinical deterioration but achieve neurological recovery. The development of multi-modal prediction models, including clinical data, imaging, and electrophysiological findings, is promising. However, it is likely to be optimized with the inclusion of new emerging diagnostic tools, such as TMS and advanced MRI techniques, and a better understanding of the biological injury.

Numerous advancements have been and are being made in the field, and ongoing research efforts promise further steps for improved patient care in DCM.
